# Gene Therapies for Monogenic Autism Spectrum Disorders

**DOI:** 10.3390/genes12111667

**Published:** 2021-10-22

**Authors:** Wout Weuring, Jeroen Geerligs, Bobby P. C. Koeleman

**Affiliations:** Department of Genetics, University Medical Center Utrecht, 3584 Utrecht, The Netherlands; w.j.weuring-2@umcutrecht.nl (W.W.); jeroengeerligs95@gmail.com (J.G.)

**Keywords:** ASD, gene therapy, gene edit, RNA therapy, epilepsy, gene replacement, gene delivery, CRISPR/Cas9, noncoding RNA, gene edit

## Abstract

Novel genome editing and transient gene therapies have been developed the past ten years, resulting in the first in-human clinical trials for monogenic disorders. Syndromic autism spectrum disorders can be caused by mutations in a single gene. Given the monogenic aspect and severity of syndromic ASD, it is an ideal candidate for gene therapies. Here, we selected 11 monogenic ASD syndromes, validated by animal models, and reviewed current gene therapies for each syndrome. Given the wide variety and novelty of some forms of gene therapy, the best possible option must be decided based on the gene and mutation.

## 1. Introduction

**Figure 1:**
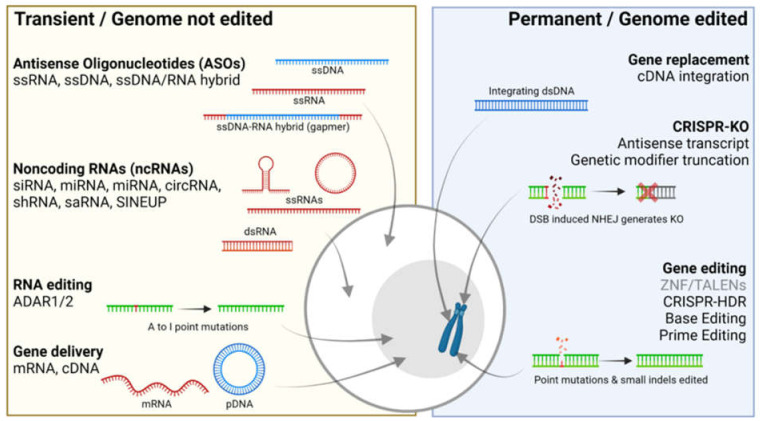
Gene therapies for syndromic autism spectrum disorders. Left; transient gene therapies such as antisense oligonucleotides (ASO), noncodingRNA (ncRNA), RNA editing and gene delivery. Right; Permanent gene therapies inlcude those that edit the host cell genome such as gene replacement (aimed at integration in the genome), CRISPR-KO (for targeted genomic disruptions) and Gene editing (for targeted repair of disease-causing mutations). Red and blue nucleotides display synthetic RNA or DNA, respectively. Green nucleotides display host-cell endogenous RNA or DNA. Created with BioRender.com.

At present, gene therapies are on the rise (Figure 1). The first approved gene therapies for human diseases are now being used in the clinic, and we are experiencing an exponential rise in putative gene therapies in development [1,2]. A substantial proportion is developed by the pharmaceutical industry, including an equally increasing number by new start-up companies, involving investments of millions of dollars. Therefore, it is expected that gene therapies will become a possibility for a large range of diseases, including autism. Most current gene therapies are focused on monogenic diseases with a strong penetrant single genetic cause that is well understood in terms of disease mechanism. For autism, the development is centered around the monogenic forms. Intensive genetic studies have confirmed that autism spectrum disorder (ASD) has a strong genetic basis that shows a high degree of genetic heterogeneity [3]. The first important distinction is that ASD can be either the sole clinical phenotype or syndromic. In the latter case, ASD is a symptom of a developmental disorder that includes multiple phenotypes, such as epilepsy, intellectual disability and dysmorphic features [4]. For non-syndromic ASD, there is genetic evidence for a polygenic or multifactorial genetic architecture, and it is expected that the risk for disease is determined by a combination of multiple environmental and genetic factors [5]. Nevertheless, single gene mutations have also been found that can explain disease in the relatively benign form of sporadic non-syndromic ASD. In contrast, syndromic ASD is typically more severe, and an underlying single genetic cause is known in most cases. It is for this category of ASD that gene therapy is most promising, and for which the first gene therapies are now in an advanced stage of development (Table 1). All known mutations that cause syndromic ASD lead to loss of protein function, meaning that therapies should aim at upregulation, enhancement or stabilization of healthy gene products in order to compensate for the mutation effect. There are several technologies that can achieve this, which we separated in two categories: (1) those that do not modify the host cell genome and (2) those that do (Figure 1). Several technologies have been tested to treat the effects of monogenic ASD mutations in a pre-clinical setting (Table 1), paving the way for gene therapies. In this review, we provide an overview of current gene therapies in development for 11 monogenic autism syndromes, which were chosen based on previous clinical publications and validation by animal models. We describe the different types and mechanisms of gene therapies and discuss how they can be applied to the different types of genetic autism.

## 2. Transient Gene Therapies That Do Not Edit the Genome

Current transient gene therapies that do not edit the genome are based on noncoding RNA (ncRNA), antisense oligonucleotides (ASO), RNA editing and gene delivery (Figure 1). NcRNA therapies involve various technologies that use different types of small RNA, including small interfering RNA (siRNA), microRNA (miRNA), short hairpin RNA (shRNA), circular RNAs (circRNA), small activating RNA (saRNA) and SINEUPs. Many of these RNAs act through the RNA interference (RNAi) pathway and aim to reduce gene expression at the transcriptional level, by binding to their pre-mRNA or mRNA targets and thus labeling them for degradation [23]. Consequently, these ncRNA-based drugs are aimed at gene silencing which it not applicable for LoF mutations in monogenic ASD, although there are a few exceptions. A possibility is to use RNAi techniques to inhibit the inhibitor. For example, regulatory genes or transcription factors that inhibit expression of the target gene can be inhibited using RNAi to increase expression of the target. A putative example exists for *SCN1A*, involved in developmental epilepsy and associated with ASD. Expression of *SCN1A* was shown to be negatively regulated by modifier genes *RACK1* and *MDH2,* and silencing of these genes led to SCN1A upregulation [24,25]. Unfortunately, little is known about gene regulation and side-effects of regulatory gene intervention for most monogenic ASD genes, providing an opportunity for further investigation. 

SaRNA, circRNA and SINEUP are more novel ncRNA technologies and aimed at increasing gene expression, although at different levels. saRNAs are small, double-stranded RNAs that can be designated to regulatory and promoter regions in the genome where they can enhance transcription by altering the chromatin state, although the exact mechanism is still under investigation [26]. CircRNA are circular single-stranded RNA molecules that can be designed to bind microRNAs, which would normally negatively regulate target mRNA. By binding to miRNA, its inhibition on mRNA is lost, leading to the upregulation of the target gene. A single circRNA can have more than 50 miRNA binding sites, allowing strong levels of gene regulation [27]. SINEUP are antisense long noncoding RNAs that bind to 5′ untranslated regions of mRNA, stabilizing the molecule and preventing it from being degraded. SINEUP thus act on the post-transcriptional level and lead to protein but not mRNA upregulation of target genes [28]. Each of these expression-enhancing ncRNAs are relatively new and no developments have been achieved for monogenic ASD genes so far.

In contrast to ncRNA, antisense oligonucleotides (ASO) are in a more advanced stage of development. ASO are noncoding single-stranded DNA, RNA or DNA–RNA hybrid molecules, less than 50 bp in size and can be designed for various mechanisms of action [29]. In the simplest approach, comparable to ncRNA, an ASO can reduce the expression of a target gene by binding to the mRNA. The mechanism of action involves the RNAse H1 machinery that catalyzes cleavage of the mRNA upon binding of an ASO ssDNA molecule. Such an approach has been designed and tested to target toxic CGG repeats in the *FMR1* gene. Healthy individuals can have several CGG repeats in this region, but when this number exceeds 55, patients develop fragile X associated tremor/ataxia syndrome (FXTAS) or fragile X syndrome (FXS) when the repeat number exceeds 200. By designing an ASO that targets toxic CGG repeats in the *FMR1* mRNA, the amount of disease-causing transcripts was reduced in vitro and in vivo [15]

A variation of this approach is used for Angelman syndrome (AG), caused by structural variants in the 15q11-13 locus, often leading to complete loss of the *UBE3A* gene, other LoF mutations in *UBE3A* or imprinting errors leading to inactivation of the maternal copy of the gene. In healthy individuals, the paternal copy of the *UBE3A* gene is imprinted, leading to a maternal-specific expression, which, in the case of AS, is abolished by LoF mutations. The imprinting of the paternal allele of *UBE3A* is regulated by a paternally expressed *UBE3A* antisense transcript (*UBE3A-AS*). Therefore, an ASO targeted to this natural antisense transcript was able to un-silence the paternal allele, restoring normal expression of *UBE3A* in vitro and in vivo [6]. The ASO that targets *UBE3A-ATS* is under development by two pharmaceutical companies and currently in phase 1 or 2 clinical trials [29].

ASO can also be designed to affect pre-mRNA splicing. A recent in silico prediction study on 8000 protein coding genes, including those covered in this review, showed that alternative splicing, which usually leads to nonproductive protein, can be prevented by ASO increasing the chance of normal splicing, thereby increasing the levels of functional protein. Several genes were validated in vitro, including *SYNGAP1*, for which mutations lead to syndromic ASD. The ASO was designed to bind the 3′ splice site between exon 10 and 11, thereby splicing out an alternative exon. Both mRNA and protein levels of SYNGAP1 were increased in vitro [22]. Finally, ASO can also act on the level of protein translation by blocking translation from alternative translation initiation sites such as upstream open reading frames (uORF). Binding to uORF by ASO blocks its access to the translation machinery, thereby favoring the use of the original ORF to increase productive protein [30]. Several other mechanisms by which ASO can be used to compensate for LoF mutations are explored, but are not yet fully studied for monogenic ASD.

The term gene delivery is used for delivery of healthy genes in the form of complementary DNA (cDNA) as extrachromosomal DNA (ecDNA), or extrachromosomal circularDNA (eccDNA) to the host cell. It is to some extend comparable to gene replacement, but is not aimed at alterations of the host cell genome. Gene delivery can theoretically be applied to all LoF mutations, but is the candidate approach for structural deletions of large size, which are the underlying cause for AG, Phelan–McDermid syndrome (PMD) and *NRXN1*-related monogenic ASD. By introducing an additional copy of the gene that is lost, the effect of the mutation can to some extend be compensated. Gene delivery has already been tested for FXS [16], AG [7], Rett syndrome (RTT) [10,11] and Tuberous sclerosis complex (TBC) [18], both in vitro and in vivo, highlighting that this type of gene therapy covers the majority of monogenic ASD syndromes. For each of the above-mentioned syndromes, cDNA of the corresponding mutated gene was delivered using recombinant adeno-associated viral vectors (rAAVs). rAAV infects the host cell with the additional gene copy, which localizes to the nucleus where it is transcribed from the cell-native transcription machinery. While the majority of rAAV-mediated delivered cDNAs reside as ecDNA in the nucleus, rare integration events occur as well, which seem to be concentrated in regions with transcriptionally active genes, CpG-islands and chromosomal breakage sites [31]. While the chance of random integration is much lower than the chance that cDNA remains in the nucleus as ecDNA, this could still pose a risk of disrupting otherwise normal functioning genes or regulatory regions. In addition, to maintain correct regulation, promoter type and perhaps even regulatory regions such as 5′ and 3′ UTR should be considered flanking the cDNA, although this is not yet standard practice. EcDNA introduced by rAAV is typically lost after several cell divisions, but can remain present in mature neurons for several years [32], meaning that for monogenic ASD, gene delivery could become a long-lasting therapeutic solution.

Finally, programmable RNA editing is a developing field that allows the correction of disease-causing mutations at the transcriptome level, leaving the genome intact. Current RNA editors are based on adenosine deaminases acting on RNA (ADAR), which allow adenosine to inosine transversions (‘A-to-I editing’) [33]. Inosine is typically recognized as guanine by the translation machinery, allowing targeted A > G editing in mRNA transcripts. For monogenic ASD, RNA editing has already been tested for RTT, which is caused by mutations in the *MECP2* gene. The R106Q mutation, which normally leads to RTT, was removed in the mRNA transcript by reversing the G > A motif at position 317 in an RTT mouse model [12]. After one month, mice treated with RNA editing showed 50% edited *MECP2* mRNA in hippocampal neurons, highlighting that this type of gene therapy is both effective in vivo an in vitro.

The above technologies have in common that the host cell genome is not edited, which may alleviate concerns about safety, but therefore require repeated dosing throughout life. Repeated dosing provides a degree of control over adverse effects but poses a significant burden for the patient. More seriously, adverse effects such as immune reactions to both viral and non-viral delivery vehicles remain a concern.

## 3. Permanent Gene Therapies That Alter the Genome

The second category of gene therapies includes those with the aim of permanently changing the host genome. Current technologies are gene replacement, gene editing and CRISPR-KO. Gene replacement aims to introduce an additional cDNA copy to compensate for LoF mutations by focusing on integrating the cDNA copy into the host cell genome to maintain a life-long stable expression. Currently, lentiviral vectors (LV) are the method of choice to deliver exogenous cDNA to the host cell. LV infection is followed by insertion of cDNA into the genome by the integrase enzyme, leading to long-term stable expression in both dividing and non-dividing cells [34]. An example of gene replacement for monogenic ASD involves the delivery and replacement of *Ube3a* by an LV in a mouse model of AS. As discussed above, AS is caused by mutations that cause loss of function of the only active maternal copy of the *UBE3A* gene. First, human haemopoietic stem cells were transduced with an LV containing the *Ube3a* gene, and subsequently engrafted in the AS mouse. Eight weeks after delivery, both neonate and adult mice showed normalized *Ube3a* levels in parallel to a phenotypic rescue [12]. As the animal model was successfully treated in both developmental stages, the therapeutic window for gene replacement for AS might be broader than initially expected. Even if the AS mutation is already present and patients are treated at later stages in life, there might still be a possibility to halt or reverse disease prognosis using LV-mediated gene replacement. Equal to the location of sporadic rAAV integration events, LV genomes are integrated at random positions in the genome and seem to be concentrated in active genes, which is undesired. Efforts to improve LV safety are ongoing and include engineered strains that do not longer integrate randomly, but only in so-called ‘safe harbor’ loci [35].

This year, the first in-human gene editing clinical trials started, highlighting that truly curative treatments are closer to becoming a reality. While initially, zinc-finger nucleases (ZFN) and transcription activator-like effector nucleases (TALENs) were the method of choice to edit genomic DNA, they are now replaced by those based on CRISPR/Cas9 [36], which offers ease of design, development and increased efficacy. The most important techniques are CRISPR/Cas9-homology directed repair (CRISPR-HDR), base editing (BE) and prime editing (PE) [37]. In brief, CRISPR-HDR was discovered in 2013 and is based on the Cas9 nuclease, which is guided by an RNA guide sequence (sgRNA) to the target genomic DNA. Here, a double-strand break (DSBs) is introduced at the mutation site, which in turn is repaired using a repair template DNA oligo with the correct edit via the homology-directed repair (HDR) pathway. Dividing cells use both HDR and non-homologous end joining (NHEJ) to repair DSBs, but in non-dividing cells, NHEJ is preferred, limiting the application window of CRISPR-HDR [38]. Moreover, due to the DSBs, and the separately introduced HDR template, CRISPR-HDR can introduce random insertions and deletions (indels) at the DSBs site, which are undesired [39]. BE also uses an sgRNA and is based on the original Cas9 enzyme but engineered to create single-stranded ‘nicks’ in the DNA (Cas9n), fused to a deaminase enzyme. Currently, cytosine BE (CBE)- and adenosine BE (ABE) are available and allow C > T or A > G conversions, respectively [38]. ABE and CBE have much higher levels of correct editing and lower indel rates due to the absence of DSBs [37]. However, the 4–7 base pair (bp) editing window can lead to additional C and A bases to change as well, called ‘bystander edits’ [40]. Bystander edits, which are off-target mutations, can be tolerated in some cases if they do not lead to amino acid changes, or they can be ruled out the editing window consisting of a G/T-rich region. PE, the most recent version of CRISPR/Cas9 discovered in 2019, also uses Cas9n, but is fused to a reverse transcriptase (RT) enzyme and allows all four edits in addition to small insertions and deletions [41]. PE uses an sgRNA just like CRISPR-HDR and BE, but is extended at the 3′ site with a RT template and called PE guideRNA (pegRNA), which carries the correct edit [42]. As the pegRNA carries both the guiding sequence and the repair template, editing efficiency is much higher than CRISPR-HDR and indel formation is reduced [38]. Given that BE and PE are relatively new, current gene editing therapies for monogenic ASD are based on CRISPR-HDR [39]. Two different mutations in MECP2 that cause RTT were corrected with CRISPR-HDR in induced pluripotent stem cells (iPSCs). Delivery was achieved via plasmid DNA (pDNA) transfection, wherein one plasmid carries the Cas9 enzyme and a second plasmid carries the sgRNA and HDR template. As pDNA transfection never reaches every cell of an in vitro population and edit efficiency of CRISPR-HDR is low, iPSCs were sorted based on fluorescence, leading to editing efficiencies of 20% for R270X [13] and 80% for T158M [14]. CRISPR-HDR was also used to correct the nonsense mutation R841X in *SHANK2* [20], which leads to monogenic ASD. Using a comparable strategy, the R841X mutation was removed in iPSCs, showing proof of principle for gene editing for monogenic ASD. While BE and PE have not yet been tested for monogenic ASD mutations, there are reports that show their potential to cure other monogenic disorders [38,42]. In one study, BE was applied on a pathogenic mutation responsible for Hutchinson–Gilford Progeria syndrome (HGP). In an HGP mouse model, BE machinery was delivered using rAAV to correct the C > T mutation. Additionally, early mice studies already show better editing precision by PE compared to CRISPR-HDR editing [43]. A challenge for CRISPR/Cas9 gene editing technologies is the size of the components, which limits delivery. While pDNA can be transfected to dividing cells to some extent and used for the creation of stable cell lines via FACS sorting, delivery to neurons is near-impossible with pDNA. Since the gene editing field is rapidly developing, researchers have found ways to overcome the size limitations, including the recently introduced downsized Cas9 and BE, which fit in a single rAAV, speeding up future gene editing efforts [44].

During the development of gene editing technologies, a simpler version of CRISPR/Cas9 was discovered. This version, using the original Cas9 enzyme and an sgRNA (CRISPR-KO) without an HDR template, can be used to introduce targeted DSBs in the genome [45]. These DSBs can be repaired by the NHEJ pathway, which lead to indels resulting in genomic disruptions [36]. In contrast to gene editing, CRISPR-KO is not aimed at repairing the disease-causing LoF mutation, but rather ‘slices out’ the mutation carrying region, resulting in indels. Two examples of CRISPR/KO usage in a therapeutic setting are mutations in regions that tolerated indels, such as intronic mutations, or gain-of-function mutations that are more severe to protein functioning than a premature stopcodon. Another possible therapeutic mechanism via which CRISPR/KO can act is to compensate for the mutation effect by disrupting other regions in the genome such as transcription factors or modifier genes. CRISPR-KO, which is much easier to achieve as there is no need for precise editing, is already applied in human patients. For example, patients with transfusion-dependent β thalassemia (TDT) and sickle-cell disorder (SCD) caused by reduced levels of y-globulin can be treated by using CRISPR-KO targeted to a transcription factor named *BCL11A* [46]. By knocking out *BCL11A*, which negatively regulates y-globulin, patients with TDT and SCD show strong allelic editing levels after treatment in combination with myeloablation and increased fetal hemoglobin, improving their clinical picture. In addition to ex vivo studies that use extracted patient cells that are reintroduced after they have been treated with CRISPR-KO, the first in vivo CRISPR-KO clinical trials have started just recently. For the first time in humans, CRISPR-KO was injected directly in the retina of Leber congenital amaurosis 10 (LCA10) patients [47]. LCA10 is caused by mutations in the *CEP290* gene, usually in exons. Some patients, however, show mutations in the intron between exon 26 and 27, which negatively affect splicing. By guiding CRISPR-KO with two sgRNAs to sites flanking the mutation, the complete mutation-carrying region can be removed, recovering correct splicing and resulting in a functional *CEP290*. The second in vivo CRISPR-KO clinical trial is aimed at knocking out a complete gene in patients with transthyretin amyloidosis (TTR). TTR is caused by misfolding of the TTR protein due to mutations in the *TTR* gene. By introducing DSBs in the coding region of *TTR*, overall TTR levels were reduced up to 28 days after transfusion [48]. For monogenic ASD, CRISPR/KO is also tested as a treatment for AG by knocking out the *UBE3A* antisense transcript (*UBE3A-ATS*), which silences transcription as outlined above. This strategy has been tested in mouse models for AG and the treatment was able to rescue the disease phenotype [9]. Finally, for FXS, CRISPR-KO was used to introduce DSBs in the toxic CGG repeat expansion, recovering *FMR1* expression in vitro, exemplifying another situation where CRISPR-KO can be employed with limited genotoxicity [17]. Still, CRISPR-KO can only be used to disrupt genomic regions, not to repair missense or truncating mutations, which are the majority of all monogenic diseases including monogenic ASD. Therefore, both CRISPR-KO and gene editing via CRISPR-HDR, BE or PE should be considered based on the patient mutation. 

## 4. Discussion

Monogenic, syndromic ASD is a severe disease that is difficult to treat, warranting novel treatment. Gene therapies that permanently alter the genome can become a game-changing novel therapy for syndromic ASD, with the potential to alleviate the disease symptoms with a few, or even single treatment. Gene therapies that have a transient effect, such as ASOs, ncRNA and RNA-editing leave the genome unedited and will require repeated dosing, but may have the advantage of being controllable and reversible. These differences highlight the important concerns of gene therapies, namely the issues of dosing, delivery and safety, which we did not discuss extensively here. Nevertheless, with the rapid pace of technological development comes improved viral and non-viral vectors including nanoparticles for delivery, more accurate gene editors that show little off-target effects, and controlled transgene expression. It is remarkable how rapidly precise gene editing of a single nucleotide gene mutation is approaching clinical application. For ASD, both transient and permanent gene therapies have already been tested in-vitro and in-vivo fast-fowarding translation to future medication. The type of gene therapy largely depends on its specific genetic cause, as structural variants require a different approach than missense mutations. Accurate DNA diagnostics and correct prediction of the functional consequences of the mutation will likely be equally important for the safe application of gene therapies in the future.

## Figures and Tables

**Table 1:** Monogenic Syndromic ASD Covered in this Review.

Syndrome	Gene	Patient Mutations	Mouse Model	Transient	Permanent
Angelman	15q11-13 (*UBE3A*)	SV; Loss of allele	*Ube3a* various	**ASO** UBE3A-ATS [6] **GD** rAAV9-*Ube3a* [7]	**GR** LV-*Ube3a* [8] **KO** *Ube3a-ATS* [9]
Rett	*MECP2*	Missense, truncating	*Mecp2* various	**GD** PHP.eB-iMecp2 [10] **GD** rAAV9-MECP2 [11] **RE** 317G > A (R106Q) [12]	**GE** R270X [13] **GE** T158M [14]
Fragile X	*FMR1*	Repeat expansion	*Fmr1* various	**ASO** CGG repeat [15] **GD** rAAV9-*FMR1* [16]	**KO** CGG repeat [17]
Tuberous sclerosis	*TSC1*, *TSC2*	Missense, truncating	*Tsc1^f/f^*, *Tsc2^f/−^*	**GD** rAAV8/9-*TSC1* [18] **GD** rAAV9-*TSC2* [19]	
Phelan- McDermid	22q13 (*SHANK3*)	SV; Loss of allele	*Shank3* various		
Not specified	*NLGN4X*	Missense, truncating	*Nlgn4^−/−^*		
	*NRXN1A*	SV; Large deletions	*Nrxn1a^−/−^*		
	*SHANK2*	Missense, SV	*Shank2* various		**GE** R841C/* [20]
	*SCN2A*	Missense, truncating, splice-site	*Scn2a^+/−^*		**GE** not specified [21]
	*CHD8*	Missense, truncating	*Chd8^+/∆SL^, Chd8^+/∆L^*		
	*SYNGAP1*	Truncating, splice-site	*Syngap1^+/−^*	**ASO***SYNGAP1* splice-site [22]	

**SV**: Structural variants **ASO**: Antisense oligonucleotide, **GD**: Gene delivery, **RE**: RNA editing, **GR**: Gene replacement, **KO**: CRISPR-KO, **GE**: Gene editing.
